# The Polyphenols as Potential Agents in Prevention and Therapy of Prostate Diseases

**DOI:** 10.3390/molecules24213982

**Published:** 2019-11-04

**Authors:** Tomislav Pejčić, Tomislav Tosti, Zoran Džamić, Uroš Gašić, Aleksandar Vuksanović, Zana Dolićanin, Živoslav Tešić

**Affiliations:** 1 Clinic of Urology, Clinical Centre of Serbia, 11060 Belgrade, Serbia; tomislav.pejcic@gmail.com (T.P.); dzamiczoran960@gmail.com (Z.D.); avuksano@mts.rs (A.V.); 2 Faculty of Medicine, University of Belgrade; Bulevar Despota Stefana 142, 11060 Belgrade, Serbia; 3 Faculty of Chemistry, University of Belgrade, Studentski trg 12-16, P.O. Box 51, 11158 Belgrade, Serbia; tosti@chem.bg.ac.rs; 4 Institute for Biological Research “Siniša Stanković”, University of Belgrade, Bulevar despota Stefana 142, 11060 Belgrade, Serbia; urosgasic@gmail.com; 5 Department for Biomedical Sciences, State University at Novi Pazar, 36300 Novi Pazar, Serbia; zdolicanin@np.ac.rs

**Keywords:** polyphenols, prostate, cancer, isoflavones, flavan-3-ols

## Abstract

In recent years, the progress of science and medicine greatly has influenced human life span and health. However, lifestyle habits, like physical activity, smoking cessation, moderate alcohol consumption, diet, and maintaining a normal body weight represent measures that greatly reduce the risk of various diseases. The type of diet is very important for disease development. Numerous epidemiological clinical data confirm that longevity is linked to predominantly plant-based diets and it is related to a long life; whereas the western diet, rich in red meat and fats, increases the risk of oxidative stress and thus the risk of developing various diseases and pre-aging. This review is focused on the bioavailability of polyphenols and the use of polyphenols for the prevention of prostate diseases. Special focus in this paper is placed on the isoflavonoids and flavan-3-ols, subgroups of polyphenols, and their protective effects against the development of prostate diseases.

## 1. Introduction

### 1.1. Human Evolution and Diet

It seems that *Homo sapiens* have not yet fully adapted to the meat diet. Namely, millions of years ago all human ancestors were herbivores. It is evidenced by anatomical features and evolutionary evidence, as well as by the diet of the closest human relatives, hominids [1].

Only some 15,000 years ago, man began to build settlements, raise livestock, and eat the meat of domestic animals. The transition to meat foods was probably related to a sharp increase in the number of free radicals in the cells and oxidative stress occurrence. At the same time, man reduced plant intake, from some 3000 species to only 20–30 species; the number of plant foods rich in antioxidants, vitamins, and polyphenols also reduced. Coffey believes that this change in diet brought an explosive jump in the occurrence of prostate cancer and breast cancer [2]. Today, there is a very noticeable difference between the high incidence of these two cancers in Europe, America, and Australia and the low incidence in regions where plant-based nutrition is prevalent, such as in Asia and the Far East. Increasing human lifespan is another factor that allows the cumulative effect of carcinogens. Namely, in the Neolithic period, the average lifespan was about 20 years, and today, it is 70–80 years [3].

How is the protective effect of plant foods on the human body explained? The basis of plant and animal cell functioning are similar biochemical processes, and are understandable, given the common origin of plants and animals. Namely, life on Earth existed for 3.5 billion years, and animals separated from plants about 670 million years ago [4]. The first animals lost their ability of photosynthesis and the synthesis of a large number of antioxidants, which they had to compensate by eating plants.

#### 1.1.1. Oxygen, Reactive Oxygen Species, and Oxidative Stress

Oxygen is an element without which life on Earth as we know it today would be impossible. Oxygen is the driver of an incalculable number of oxidative chemical reactions that occur in the entire living and non-living world. The first amounts of oxygen in the ocean began to produce cyanobacteria through photosynthesis, about 2.7 billion years ago, while significant oxygen production began 2.5 billion years ago, when oxygen was released from the sea and entered the atmosphere. The large amount of oxygen enormously increased the energy sources necessary for living beings, which led to the expansion of evolution [5].

The first terrestrial plants appeared 410 million years ago, some 100 million years before the appearance of the first terrestrial herbivores [6,7]. These plants found themselves in an environment rich in solar energy, which allowed more intense photosynthesis, but also the production of large quantities of reactive oxygen species (ROS). Plant cells used ROS to defend against pathogens; however, as an adaptation to the excessive and potentially dangerous amounts of ROS, the plants began to increase production of antioxidants, vitamins, and polyphenols.

#### 1.1.2. Generation of Reactive Oxygen Species in Cells

In all aerobic organisms, the energy for biological functions is generated in the mitochondria. As byproducts, ROS are also produced on a daily basis; the most important ROS are hydrogen peroxide (H_2_O_2_), hypochlorous acid (HClO), and the free radicals: hydroxyl radical (OH^−^) and superoxide anion (O_2_^−^). The largest producers of ROS are NADPH oxidase enzymes, which are found on the cell membrane, mitochondria, and endoplasmic reticulum.

Mitochondria convert the energy into a usable form, adenosine triphosphate (ATP). The process of ATP formation involves the transport of hydrogen ions (H^+^) through the inner membranes of the mitochondria, by the electron transport chain process. During this chain of reactions, electrons undergo a series of proteins via oxidative-reduction reactions; each protein in the series that receives an electron has a greater reduction potential than the previous one. The ultimate destination of the electrons in this chain is the oxygen molecule, O_2_. Under normal circumstances, O_2_ is reduced and produces water, H_2_O. However, in 0.1–2% of electrons passing through the chain, O_2_ is reduced prematurely and incompletely into radical superoxide, O_2_. Hydrogen peroxide, H_2_O_2_, is formed from superoxide, which leaks from the mitochondria. The enzymes catalase and superoxide dismutase convert H_2_O_2_ to oxygen and superoxide to H_2_O_2_, from which H_2_O is formed. However, this conversion is not 100% efficient and a certain amount of H_2_O_2_ lags in the cell. If too much damage occurs in the mitochondria, programmed death, apoptosis, occurs in the cell. The Bcl-2 protein, located on the surface of the mitochondria, detects damage and activates a class of protein called Bax, which drill holes in the mitochondrial membrane and leads to the leakage of the protein of the electron transport chain, called cytochrome C. After that, the released cytochrome C begins a series of reactions leading to the cleavage of proteins in the mitochondrial membrane and its disintegration [8].

ROS is considered—when formed as by-products of normal oxygen metabolism—to have an important role in cell signaling and homeostasis. Small, normal amounts of ROS generated within a cell initiate a lot of important processes, such as regulation of apoptosis, induction of various defense genes, release of ROS from platelets during hemostasis, leukocyte attraction, activity during an inflammatory response, etc. Under normal circumstances, the cell is able to counteract the effects of smaller amounts of ROS by producing antioxidant molecules. However, if the amount of ROS exceeds the antioxidant capacity of the cell over time, the genetic material and other parts of the cell can be damaged. The cumulative effect of excessive amounts of ROS is called oxidative stress (OS); OS is associated with the aging and development of various diseases. There is evidence that animals that have been genetically engineered to remove antioxidant enzymes have a shorter life span [9].

One of the main causes of mutations is DNA oxidation. It is estimated that the normal daily endogenous production of ROS in a single cell modifies about 20,000 DNA bases [10]. However, during OS, the cell is unable to eliminate a large amount of ROS; ROS then activates a variety of transcription factors, leading to the synthesis of proteins that control inflammation, survival, proliferation, and invasiveness of tumor cells. It is considered for ROS to have a similar effect on normal and malignant cells: Low levels of ROS favorably affect the survival of the malignant cell, while a high level of ROS suppresses tumor growth and induces apoptosis. Ionizing radiation and cytostatics increase ROS formation [11].

Antioxidants react with ROS by self-oxidizing and thus reducing the possibility of oxidative damage to cellular organelles. The function of the antioxidant system is not to completely remove the oxidants, but to maintain them at the optimal level. Antioxidants are generally reducing agents, such as thiols, ascorbic acid or polyphenols. Human cells can produce some antioxidants on their own, like superoxide dismutase, catalase, and glutathione peroxidase, while some have to be ingested through food, like vitamin E, vitamin C, polyphenols, carotenoids, etc. [12]. When vitamins A, C, and E were identified as antioxidants, the study of them began in the biology of living beings. The early research, dealing with the way that vitamin E prevented peroxidation of lipids, represented an introduction into antioxidant studies.

## 2. Prostate and Prostate Diseases

The prostate is an organ that belongs to the accessory of the male reproductive organs. In all mammals, the prostate is involved in the production of seminal plasma, which serves as a medium for the transport and nourishment of sperms. The prostate function is controlled by steroid hormones and its structure is basically similar in all mammals. In most primates, the prostate is composed of two parts, the cranial and caudal prostate; in humans, these two parts are located in one compact organ and correspond to the central zone (CZ) i.e., peripheral zone (PZ) [13].

The diseases of the prostate are rare in mammals. Benign prostatic hyperplasia (BPH) occurs in humans, chimpanzees, and dogs. Prostate cancer (PCa) is extremely rare in all mammals, including chimpanzees and apes. The low incidence of PCa in hominids is primarily explained by the fact that they are obligate herbivores [14].

### 2.1. Prostate Physiology

The prostate structure is made up of connective stroma and epithelial cells. The stroma consists of the basic substance, axons of nerve cells and stromal cells, fibroblasts, endothelial cells, and smooth muscle cells. The prostate epithelium is composed of secretory, neuroendocrine, basal, and intermediate cells.

The most important physiological functions of the prostate are secretory and hormonal. The prostate synthesizes and secretes numerous non-peptide substances, such as polyamines, cholesterol, lipids, citrates, and zinc. Citrates and zinc are found in the prostate at a much higher concentration than in other organs and tissues [15]. The most important proteins in the prostatic secretion are prostate-specific antigen (PSA), glandular kallikrein (hK2), and prostatic acid phosphatase (PAP). The most important enzyme in the semen fluid, PSA, exerts its physiological function only in the vagina, where it cleaves the proteins of the seminal coagulum and thus enables the active mobility of the sperms. PSA molecules constantly leak from the prostatic acini in the urethra, wherefrom they are flushed during the urination act [16,17,18].

The hormonal function of the prostate is related to the intracrine and paracrine activity. Paracrine factors, acting from neighboring cells, are tissue growth factors (GFs), small peptide molecules that stimulate and/or inhibit cell division and differentiation. The balance between proliferation and cell death is dictated by the complex interactions between GFs and steroid hormones. Fibroblast GF (FGF), vascular endothelial GF (VEGF) and insulin-like GF (IGF) are thought to promote cell proliferation in BPH, and DHT enhances their action [19]. Transforming GF-β (TGF-β1) inhibits epithelial cell proliferation while stimulating fibroblast mitosis. Increased expression of TGF-β1 on stromal cells is associated with an increase in the stromal compartment [20].

### 2.2. Sex Hormones in Prostatic Tissue

**Figure 1:**
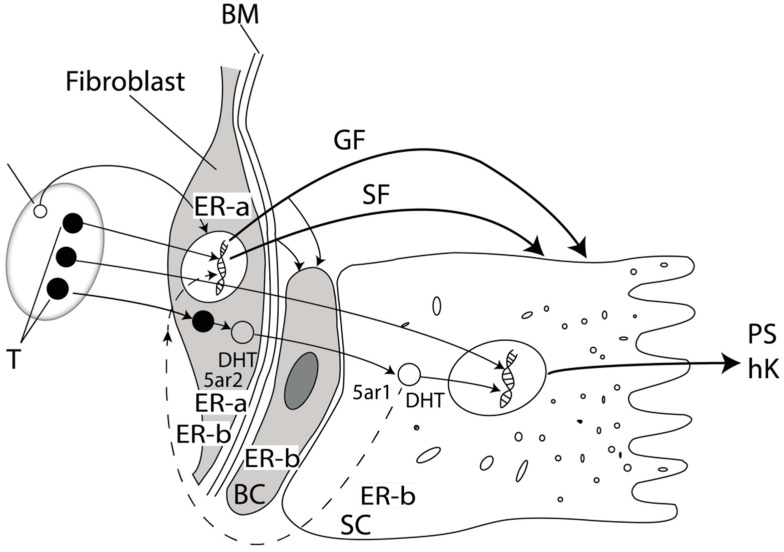
Stroma–epithelial interaction. E = estrogen, T = testosterone, DHT = dihydrotestosterone, BM = basement membrane, GF = growth factor, BC = basal cell, SC = secretory cell.

The most important serum androgen, testosterone (T), acts as a prohormone: In prostate cells, most of T is converted to potent DHT by the action of enzyme 5-alpha reductase (5-αR); a number of T molecules are converted to estradiol (E2) by the action of enzyme aromatase. Estrogens act on the human prostate via interaction with two receptor subtypes, ERα and ERβ. The dominant estrogen receptor in the prostate is ERβ, which is found both in the stroma and in the epithelium (Figure 1), whereas ERα is found primarily in the stroma. In general, ERα stimulation stimulates cell proliferation, whereas ERβ stimulation counteracts these effects [21]. The activation of ERβ influences azote oxide metabolism (NO) and thus leads to vasodilatation.

Growth factors (GF) are small peptide molecules that stimulate, and sometimes, inhibit the division process and cell differentiation, by activating cell receptors for GF. The balance between proliferation and cell death dictate the complex interactions between GF and steroid hormones. It is considered that FGF (fibroblast GF), VEGF (vascular endotel GF) and IGF (insulin-similar GF) stimulate cell proliferation with BHP, and DHT increases their effect. The most important factor is the interaction of stroma and epithelium is FGF-7 [19]. The factor of TGF-β1 (transforming GF-β) performs a line of important functions: It stimulates mitosis with fibroblast and inhibits proliferation of epithelium cells. The increased effect of TGF-β1 stroma cells is connected to the increase of the stroma section [20].

There are numerous reciprocal reactions between the stroma and the epithelium, called the stroma–epithelial interaction; this process begins during embryonic development, continues during prostate development, and lasts throughout adulthood and old age [22].

### 2.3. Prostate Diseases

Prostate diseases are of great importance in modern men: BPH is ranked fourth in the most common chronic diseases and eighth in the cost of their treatment. Prostate cancer (PCa) is the fourth most common cancer in the world and the second most common cancer in men. In the USA, PCa is the most common malignancy in men and accounts for 27% of all cancers [23,24]. Prostatitis is the third most common urological diagnosis with people of 50 years and older, after BHP and PCa, it is the most common urological diagnosis with people younger than 50 years.

### 2.4. Benign Prostatic Hyperplasia

By definition, BPH is a histologic diagnosis, which involves the uncontrolled proliferation of the prostatic stroma and epithelium and enlargement of the prostate gland. Lower urinary tract symptoms (LUTS) very often accompany BPH and enlargement of the prostate and they gradually worsen over time. The most frequent LUTS are very difficult urination and the feeling of incomplete bladder vacating, frequent urination, night urination, etc. A complete discontinuation of an urination process and a bladder stone are indications to have BPH surgery.

Global BPH prevalence is 26%. The prevalence increases with aging: After 50 years of age, it is 50%, and at the age of 60, 60% of men have BPH with LUTS. The prevalence of BPH is higher in Chinese people living in the USA than in Chinese people in China [25,26,27]. These facts are in favor of the theory that for BHP to appear, lifestyle and dieting, not only genetic heritage, are of importance.

Risk factors for BPH development are low physical activity, obesity and metabolic syndrome. The familial form of BPH is present in 50% of patients under 60 years of age who underwent simple prostatectomy [28]. The presence of androgens during the development of the prostate, puberty, and aging represents the fundamental condition for the development of BPH.

However, the role of androgens in the onset of BPH is not entirely clear, as BPH develops in elderly people with normal or low serum androgen levels. However, DHT levels in prostate tissue remain high [29,30]. Pejčić et al. proved that T and DHT accumulate in the prostatic stroma in BPH and that there is a linear correlation between T and DHT concentration in the tissue and prostate size [31]. Estrogen accumulation has also been demonstrated in prostatic tissue [32,33].

### 2.5. Prostate Cancer

Prostate cancer (PCa) is generally diagnosed in men over 65, and very rarely in the ones under 50. Today, PCa is the most commonly diagnosed cancer in men in America, Europe, Australia, and Sub-Saharan Africa [34,35]. In the USA, the highest PCa incidence is found in Afro-Americans (220), followed by white-Americans (139), and the lowest in Asian-Americans (75) [24].

Pursuant to the data of the CDC-National Program of Cancer Registries and National Cancer Institute Surveillance, Epidemiology and End Result Program in the USA, the incidence of PCa in 2014 was around 100 for all races, 50 for the Asians living in the USA, 90 for white-Americans and 150 for Afro-Americans.

PCa incidence in Asia is 50–100 times lower than in western countries. Pursuant to the data of WHO from 2012, PCa incidence in Asia was 10.5% [36]. The lowest incidence measured was in rural China (2.6) and in North Korea and Mongolia (2–3), while in Japan, it was 31 [37]. PCa incidence with the inhabitants of Shanghai was 26 times lower than with Americans [35]. Asians migrating to other countries show very fast the same epidemiological pattern. The risk of PCa development grows within the first generation of immigrants from Japan and China to the USA, probably for their transition to the western style of dieting [38,39].

Genetic factors are very important in PCa development: if the father had PCa, the son’s risk is 2× higher; if both father and brother had PCa, the risk is even 5 times higher [40]. A significant number of PCas are caused by inflammation caused by infection, nutrition or other agents. Western diet, which involves high intakes of red meat, fat, and low intake of plant foods, is one of the reasons for the highest PCa incidence in the West [41]. Obesity is an important risk factor for PCa. In obese men, there is an increased concentration of E2, insulin, free IGF-1, and leptin in serum, and a reduced concentration of free T and adiponectin. Although serum leptin levels are elevated, there is resistance to leptin in obese people. The leptin from adipose tissue is thought to play a role in the development of advanced PCa [42].

Androgens are essential for the development, growth, and survival of PCa. Castration is known to lead to apoptosis of malignant cells and the decrease of PCa bone metastases. Like in normal prostatic tissue, in PCa cells estrogens play an important role: activation of ERα stimulates proliferation, and activation of ERβ leads to an antiproliferative and pro-apoptotic effect. Over time, loss of ERβ occurs in PCa, leading to disease progression. Animal experiments show that PCa occurs in mouse strains lacking the ERβ receptor (betaERKO), but does not occur in mice lacking the ERα receptor (alphaERKO). Also, mice strains lacking the enzyme aromatase (ArKO) have a lower PCa incidence [43,44]. Therefore, for PCa prevention, the blockage of ERβ is needed and ERβ stimulation.

Vitamin D has a protective effect on the development of PCa, and men with vitamin D deficiency are known to have a higher PCa incidence. This category includes the elderly, people from the north and African-Americans; in the latter, a large amount of melanin in the skin reduces the production of active vitamin D. The vitamin D receptor, to which serum vitamin D binds, is present in the normal and malignant prostate epithelium [45]. Normal prostate cells can make a synthesis of D vitamin, and then, it inhibits their development [46].

#### 2.5.1. Cellular Signaling Pathways in Prostate Cancer

Disorders at the level of cellular molecular signaling pathways play a key role in the development of malignant tumors. In PCa, disturbances of the PI3K/Akt/mTOR signaling pathway are of great importance [47]. The major proteins of this pathway are PI3K (Phosphatidylinositol 3-Kinase) and Akt (Protein kinase B). The mTOR (mechanistic target of rapamycin) enzyme is named after rapamycin, which inhibits mTOR and thus acts immunosuppressive and antiproliferative. The PI3K/Akt/mTOR pathway regulates cell dormancy and proliferation and it is necessary for promoting the growth and proliferation of adult stem cells. In many cancers, this pathway is overactive, due to increased activity of PI3K, or Akt. Increased mTOR activity activates proliferation while decreasing tumor apoptosis [48,49].

PI3K/Akt activity is enhanced by EGF, IGF-1, and insulin. On the other hand, the natural inhibitor of PI3K/Akt, the enzyme PTEN (tumor suppressor phosphatase and tensin homolog), reduces the activity of this pathway by interfering with PI3K and negatively affecting mTOR signals. One of the most common reasons for mTOR activation is mutations or deletions in PTEN, which are present in over 80% of PCa [50].

**Figure 2:**
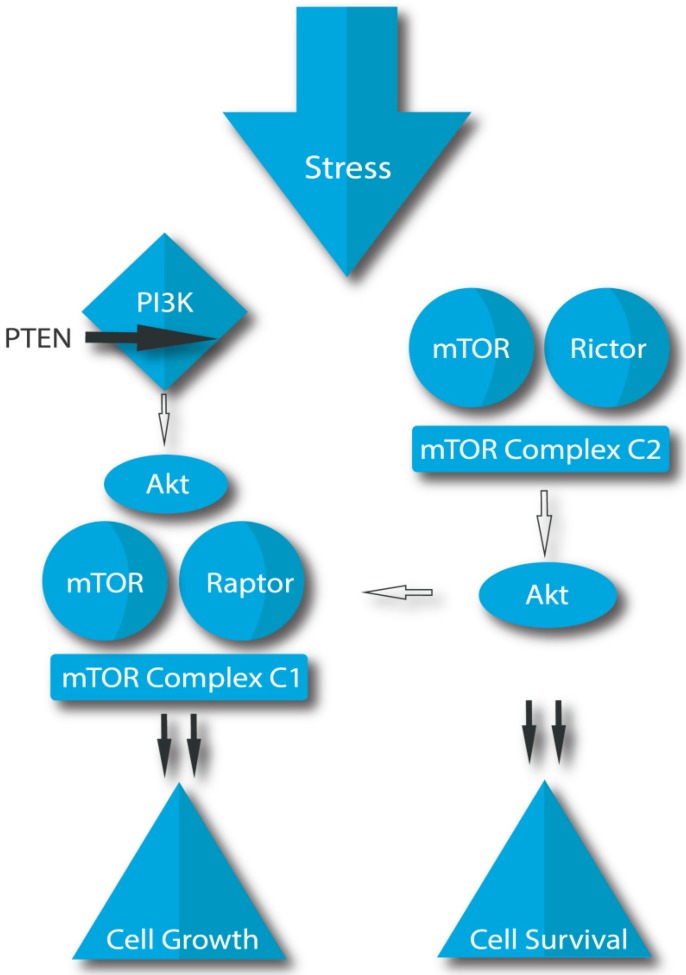
Simplified graphical presentation of the PI3K/Akt/mTOR signaling pathway and tumor suppressor phosphatase and tensin homolog (PTEN) actions.

There are two complexes created by mTOR: mTOR complex 1 (MTORC1) and mTOR complex 2 (MTORC2). The MTORC1 is the most important regulator of cell growth, responding to and integrating different signals from food and the environment; this complex is inhibited by rapamycin. The MTORC2 stimulates cell survival via Akt activation (Figure 2). Aberrant mTOR signals are responsible for the development of many diseases.

The key genetic events in PCa progression are loss of functional androgen receptor (AR) and increased expression of epidermal growth factor receptor, erbB. Genetic alterations lead to feedback between the membrane receptor (erbB1) and the associated ligand (e.g., TGFα). The result is the enhanced activation of the most important component for uncontrolled PCa growth, namely ERK1/2 (extracellular signal-regulated protein kinase 1/2) [51].

PCa resistance to therapy is associated with abnormally increased expression of AR, Bcl-2, erbB, HER2 (human epidermal growth factor receptor 2), cyclin D1, and cyclooxygenase (COX-2), all of which are associated with the activation of transcription nuclear factor NF-κB. The NF-κB is a protein complex that controls DNA transcription, cytokine production, and cell survival. NF-κB is found in the cells of almost all animals and is involved in the cellular response to stress, cytokines, free radicals, heavy metals, ultraviolet radiation, oxidized LDL, as well as bacterial and viral agents [52].

#### 2.5.2. In Vitro and In Vivo Experiments on Prostate Cancer

Human prostate adenocarcinoma cell lines, such as LNCaP, DU-145, and PC-3, are most commonly used in in vitro experiments. Androgen-dependent lines are LNCaP, obtained from PCa metastases to lymph nodes and 22Rv1. Androgen-independent lines are DU-145 and PC-3, which have no AR and have ER. Animal experiments are most commonly performed on specific strains of mice and rats, such as TRAMP (transgenic adenocarcinoma of the mouse prostate), betaERKO (mice lacking the ERβ receptor), alphaERKO (mice lacking the ERα receptor), and ArKO (mice lacking aromatase). A nude mouse is a laboratory mouse of the thymus-free variety that has an inhibited immune system due to reduced T-cells. He got his nickname because he has no hair. This variety of mice can receive different tissues and tumor grafts, without rejection [44].

### 2.6. Prostatitis

Prostatitis is the most common urological diagnosis in people under 50 and the third most common urological diagnosis in people over 50 years, after BHP and PCa. The overall prevalence of chronic prostatitis and chronic pelvic pain syndrome is 7.1%. However, the prostate inflammation is seen in as many as 44% of the prostate tissue samples at autopsy, and even in individuals who have not had prostate disease. Acute bacterial prostatitis is a prostate infection, most commonly caused by gastrointestinal gram-negative bacteria. The risk factors for prostatitis are intraprostatic ductal reflux, phimosis, indwelling urethral catheter, transurethral surgery, and secretory prostate dysfunction. Secretory dysfunction is accompanied by decreased levels of fructose, citric acid, acid phosphatases, Zn, Mg, and Ca and an increase in pH, levels of inflammatory proteins and LDH-5 / LDH-1 ratio [53].

## 3. Polyphenols

### 3.1. Occurrence in Nature

Polyphenols are the most abundant and most examined secondary metabolites in the plant kingdom, with over 8000 species known, but are still an area of research that claims many scientists [54]. Polyphenols play a key role in the growth, regulation, and structure of plants and vary between different species. Stress and growth conditions modify the structure and content of polyphenols [55]. About 2% of all carbohydrates created during photosynthesis are converted to flavonoids and their derivatives [56]. Polyphenols are found in a large number of plants, such as vegetables (artichokes, cabbage, broccoli, asparagus, avocado, beetroot, and spinach), nuts (nuts, hazelnuts, pistachio, almonds, Indian nuts, peanuts, pecans, and macadamia), dark pigmented fruit (cranberry, blueberry, plums, blackberries, raspberries, strawberries, currants, figs, cherries, guava, oranges, mangoes, grapes, and pomegranate), spice plants (cloves, cinnamon, oregano, cumin, turmeric, parsley, basil, curry powder, mustard seeds, ginger, pepper, chili powder, pepper, garlic, coriander, onion, and cardamom), and medical herbs (sage, thyme, marjoram, tarragon, mint, oregano, salty basil, and dill) [57,58].

The average daily intake of polyphenols in the European countries differs a lot: it is the highest in Denmark (1700 mg) and the lowest in Greece (660 mg). The majority of ingested polyphenols are phenolic acids (~55%), and in some regions, flavonoids (50–60%). The most important sources of polyphenols in foods are coffee, tea, and fruits [59].

### 3.2. Classes of Polyphenols

All plant phenol compounds originate from amino acids phenylalanine, i.e., silimatic acid. Silimatic pathway is a metabolic pathway, one of seven steps used by bacteria, fungus, algae, and the bio-synthesis of plants for flavonoids and aromatic amino acids, while animals and people must intake them through food. Polyphenols are classified in a number of ways, usually based on the number of phenolic rings and they also contain the elements binding these rings to each other. The major classes of polyphenols are flavonoids, phenolic acids, stilbenes, and lignans. Polyphenols can also be divided into flavonoids and non-flavonoids [60,61].

### 3.3. The Action of Polyphenols in the Body

The antioxidant properties of polyphenols have been demonstrated in vitro, in numerous experiments on isolated cell cultures; on the other hand, but a much larger problem is to prove these properties in vivo. In addition to its direct antioxidant effect, polyphenols also act indirectly in humans by increasing the level of uric acid. However, the “antioxidant hypothesis” does not offer precise answers to understand the molecular mechanisms behind the beneficial effects of polyphenols from food. Yet, the signaling effects of polyphenols are thought to occur at much lower concentrations than those required for antioxidant activity [62,63].

The second effect of polyphenols is the modification of cellular signals and the regulation of the action of various enzymes, such as PI3K, Akt/protein kinase B, tyrosine kinase, protein kinase C, mitogen-activated protein kinase, etc. Signal effects of polyphenols probably appear with a lot lower concentrations from the ones needed for antioxidant activity. In accordance with this, the “antioxidant hypothesis” does not offer true answers for the understanding of molecular mechanisms that are in the background of favorable polyphenol effects from foods. Also, another important effect of polyphenols is to increase the secretion of paraoxonase, an enzyme that prevents low-density lipoprotein (LDL) oxidation [64,65].

### 3.4. Resorption of Polyphenols from Foods

The debate regarding the absorption of polyphenols from food is still ongoing. On the one hand, epidemiological studies agree that there are positive health effects of taking polyphenols; on the other hand, there is a low bioavailability of polyphenols in the body, due to low resorption and rapid transformation and excretion. The absorption of polyphenols via the stomach and small intestine is only 5–10% of the total intake. To be absorbed, polyphenols must be chemically transformed by the intestinal microflora (“microbiota”) and enzymes from the intestinal epithelium. The absorption of polyphenols depends on the amount and size of the phenolic compound, previous diet, nutrient matrix, sex, and gut microflora [66,67].

The accurate assessment of the physiological effects of polyphenols from foods is very complicated because single foods often contain a lot of different polyphenols, whose outcome is impossible to track and measure. For example, there are about 60 different flavonoids in red wine. On the other hand, mixtures of different polyphenols can have a synergistic and antagonistic effect. Also, polyphenols effect antagonistically in the presence of vitamin C. The more powerful a polyphenol is, an antioxidant regenerates faster, while the weaker antioxidants are spent [68,69].

### 3.5. Flavonoids

Flavonoids belong to the class of low molecular weight phenolic compounds and are widespread in the flora world. They got their name from the Latin word *flavus*, which means yellow. Flavonoids are found in large quantities in fruits and vegetables and drinks such as tea, cocoa, and wine. Today, there are about 6000 flavonoids, which are mostly found as multicolored pigments of fruits, vegetables, and medicinal plants [70].

The antioxidant activity of free flavonoids depends on the number and position of their hydroxyl groups. The basic structure of flavonoid compounds is the flavone nucleus, which consists of two benzene rings (A and B) that are linked via a heterocyclic pyran C ring. The position of the benzenoid B ring is the basis for dividing the class of flavonoids into flavonoids (2-position) and isoflavonoids (3-position) [71].

The average intake of flavonoids in the USA adults is about 250 mg per day, with flavan-3-ols accounting for 80% of total intake. Tea is the main primary source of flavonoids and accounts for 80% of the intake of all flavonoids. In Korea, the average daily intake of flavonoids is about 110 mg [72,73]. The main sources of flavonoids are in bio-kimchi (the Korean food of fermented vegetables), green tea, Japanese apples, and soya.

The medical use of flavonoids is based on their antioxidant, anti-inflammatory, and anticarcinogenic properties and their capacity to modulate key enzymatic functions in the cell. They are strong inhibitors of enzymes such as xanthine oxidase, cyclooxygenase, lipoxygenase, and PI3-K. The primary role of flavonoids in plants is to protect them from UV radiation, living or inorganic stress, etc. In the food industry, flavonoids are added to foods to prevent fat oxidation.

There are numerous classifications of flavonoids. According to the degree of oxidation of the central piran ring, flavonoids can be divided into six classes: 1. flavones, 2. flavonols, 3. flavanones, 4. isoflavonoids, 5. flavan-3-ols (monomers and oligomers), and 6. anthocyanins.

#### 3.5.1. Flavones

**Figure 3:**
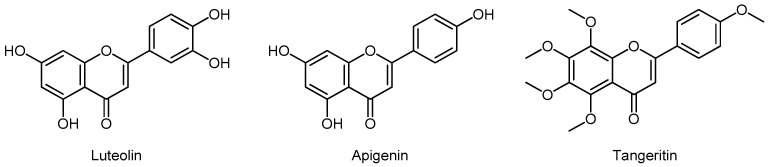
Chemical structures of luteolin, apigenin, and tangeretin.

Flavones are widely represented in leaves and flowers. The most important sources of flavones are celery, parsley, red pepper, chamomile, mint, and ginkgo biloba. Their well-known representatives are luteolin, apigenin, and tangeretin (Figure 3).

Han (2018) found that luteolin inhibited PC-3 cell proliferation, migration, and cell renewal [74]. Apigenin showed similar activity on LNCaP and PC-3 lines. In a study in TRAMP mice, apigenin was shown to suppress PCa formation via the PI3K/Akt/FoxO signaling pathway. There was a decrease in tumor size and disappearance of distant metastases in TRAMP mice which received apigenin for 20 weeks [75]. Tangeretin induced epithelial–mesenchymal transition in PC-3 cells, via the PI3K/Akt/mTOR signaling pathway [76].

#### 3.5.2. Flavonols

**Figure 4:**
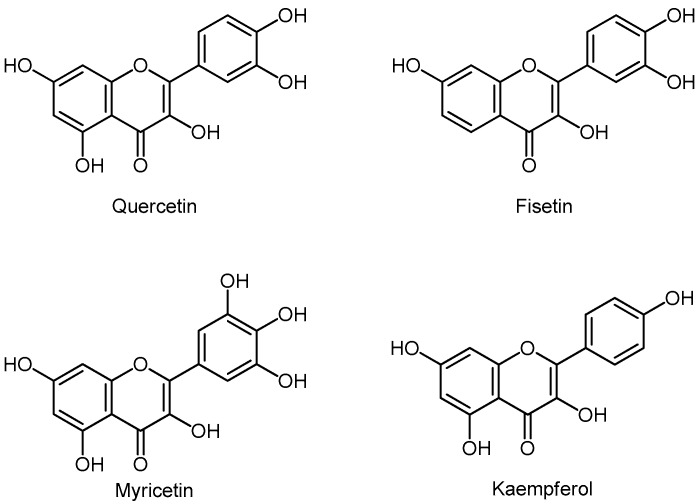
Chemical structures of quercetin, fisetin, myricetin, and kaempferol.

The best-known flavonols are quercetin, fisetin, myricetin, and kaempferol (Figure 4). Flavonols are present in onions, kale, lettuce, tomatoes, apples, grapes, and berries, as well as in tea and red wine.

Quercetin is largely found in red grapes, red wine, apples, tea, and berries. The highest concentration of quercetin is found in canned capers (~170 mg/100 g) and red onions (33 mg/100 g). Quercetin acts as an antioxidant, as a protein kinase inhibitor and activator of ERα and ERβ. The action of quercetin is three times weaker than E2; however, it is nine times more selective for ERβ, than for ERα [77]. The FDA states that quercetin is neither an antioxidant, nor a deficient nutrient, nor a cure for any disease in humans. Nevertheless, Paller (2015) found that in African Americans with vitamin D deficiency increased the intake of quercetin leads to a decreased risk of PCa. Sun (2018) states that quercetin in combination with metformin inhibits growth, migration and invasion on PC-3 and LNCaP cells by strongly inhibiting the VEGF/Akt/PI3K signaling pathway [78,79].

Fisetin is found in apples, grapes, kiwi, strawberries, onions, and cucumber. On the PCa cell lines, fisetin has been shown to act as a dual inhibitor of PI3K/Akt and mTOR metabolic pathways. This is an important finding, as mTOR activation is a lot more common in tumors with high PI3K/Akt expression. It is believed that fisetin could be probably used in the treatment of PCa, alone or in combination with chemotherapeutic drugs [47].

Kaempferol showed cytotoxicity across different cancer cell lines including Du145; it is offered as a potential anticancer agent [80].

#### 3.5.3. Flavanones

**Figure 5:**
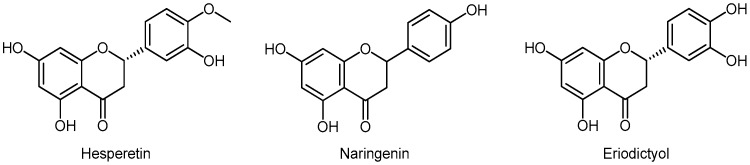
Chemical structures of hesperetin, naringenin and eriodictyol.

Flavanones are present in citrus fruits, oranges, lemons, and grapes. The most well-known are hesperetin, naringenin, and eriodictyol (Figure 5).

Hesperetin inhibits proliferation and induces cell cycle arrest in PC-3 cells, in the G1 phase. Similarly, naringenin, natural flavonoid from grapefruit, and fruit, inhibits proliferation and migration and induces ROS production and apoptosis on PC3 and LNCaP cells. Naringenin has been shown to enhance the effect of paclitaxel on reducing progression in PCa lines [81,82].

#### 3.5.4. Isoflavonoids

**Figure 6:**
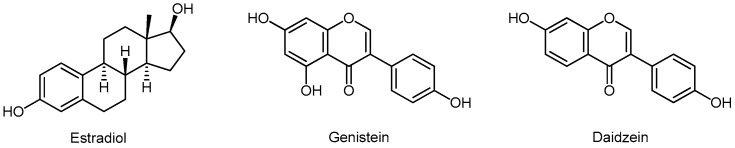
Chemical structures of estradiol, genistein, and daidzein.

Isoflavonoids are a large and inhomogeneous group of flavonoids. Isoflavonoids are derived from the flavonoid biosynthesis pathway, via liquiritigenin or naringenin. In plants, they play a role in the formation of phytoalexins during defense against germs. The most important isoflavonoids belong to the subgroup of isoflavones and these are genistein, daidzein, and glycitein (Figure 6) and their glycosides, genistin, daidzin, and glycytin. Unlike flavonoids, in which the benzenoid B ring is in position 2, in the isoflavonoids the B ring is in position 3. The chemical structure of genistein and daidzein is very similar to 17b-estradiol, E2 (Figure 6). These two isoflavones are also similar to each other and differ in only one hydroxyl group, and share a common diphenolic structure. Genistein competes with E2 for binding to the ER; however, genistein has more than 300-fold higher binding affinity for ERβ than for ERα [83].

The largest source of isoflavones in nature is soya, which contains about 200 mg of isoflavones per 100 g of fresh product. Genistein and genistin make 30–60% of total soya isoflavones, daidzein, and daidzin 40–60%, while glycitein and glycitin make 1–13% of total soya isoflavones. Genistin and daidzin in soya are in conjugated form with sugars, as glycosides. Glycosides are converted into active forms, genistein, and daidzein, by fermentation, which is the traditional way of preparing soya in Asia [84]. Except in soya, genistein and daidzein are also found in beans, peas, legumes, and peanuts, but in significantly smaller quantities.

In soybean flour, the level of genistein is lower than genistin about 50 times, but only two times in fermented soya products. Specifically, during microbial fermentation, the beta-glycosyl bond of genistin is cleaved and genistein is formed [85]. A fermentation-like process occurs in the jejunum, in the intestinal wall, where the biologically inactive forms are hydrolyzed by the action of bacterial enzymes, β-glucosidases, and converted to biologically active aglycones, daidzein and genistein, which can be absorbed by intestines. The intestinal flora can convert daidzein into several products, including isoflavonoids equol and dihydrodaidzein. Interestingly, only 33–50% of people have a suitable bacterial strain that can produce the estrogen metabolite equol. These individuals, called “equol producers,” have more abilities to get estrogenic effects from soya. After their intake, the highest serum isoflavone values appear after 4–6 h, have a half-life of 4–8 h, and almost all isoflavones are excreted after 24 h [86,87].

The average daily isoflavonoid intake ranges 0.3–0.8 mg in Europe and the USA and 50–100 mg in Asia. Vegetarians and vegans in Europe consume an average of 22 mg of food isoflavones and 50 mg of supplements. In some epidemiological studies, it was determined that an average daily intake of isoflavones in Japan and China is up to 75 mg and even 150–200 mg in the traditional Japanese diet [84,88,89]. In different studies, the average intake of genistein and genistin is estimated to be roughly 8–12 mg daily and 30 mg daily.

The average plasma isoflavones concentration in Europeans is 0.01 μmol/L and around 0.6 μmol/L in Japan [85,88]. Isoflavones are specifically concentrated in prostatic fluid, in a concentration several times higher than in plasma, especially in people who consume soya products [90]. In prostatic tissue, genistein levels were the lowest in PCa (8.4 ng/g) than in large volume BPH (8.8 ng/g), and the highest in small volume BPH (20.9 ng/g tissue) [91].

##### Bioavailability of Isoflavones

Genistein, like all other bioactive phytochemicals, has two major disadvantages: low bioavailability and high pleiotropy. The oral bioavailability of genistein is lower than that of genistin, due to its poor solubility in water. The plasma concentration of genistein from food is much lower than the concentration required for initiating an anticancer response in an experimental model. A significant clinical effects of genistein can be expected when given at pharmacological doses of >100 μM/L and weak effects when it is given at chemopreventive doses <1 μM/L. Pleiotropy is a phenomenon when a compound acts at several levels in a cell and at the same time initiates several biochemical reactions; the anticancer effects of genistein are probably the result of numerous synergistic mechanisms [92].

##### Estrogenic and Antiestrogenic Activity of Isoflavones

Isoflavones are called plant estrogens because of their structural similarity to human estrogens and their mild estrogenic activity. Compared to physiological estrogens, isoflavones have only between 0.01% and 0.1% of E2 activity on the molar base. However, isoflavones exert physiological effects in high serum concentrations. Studies have shown that the concentration of isoflavones in people who eat soya is at the micromole level and in people who do not eat soya, at the nanomole level [93].

A very important characteristic of isoflavones is that they do not have the same binding affinity for both estrogen receptors. Specifically, isoflavones have a much higher binding affinity for ERβ than for ERα; the relative binding affinity of genistein for ERβ is 36% and for ERα, 5% [94]. In various studies, it was determined that genistein has a 40- to 60-fold greater affinity for ERβ than for ERα, and daidzein, 5-fold. However, the genistein–daidzein–equol mixture has much higher selectivity for ERβ than for ERα (82.6), suggesting their potential synergistic interactions. Isoflavones activate ERα in, the absence of circulating estrogens, but competitively inhibit it at high estrogen concentrations [95,96].

Kostelac and co-authors found that half-maximal effective concentration, EC (50) for ERα activation, for E2, genistein, and daidzein, were 0.03 μM, 15 μM, and 300 μM, respectively. On the other hand, the EC (50) for ERβ activation, for E2, genistein and daidzein, were 0.01 μM, 0.03 μM and 0.35 μM, respectively [97]. Receptors ERβ are predominant in the prostate; in benign and malignant prostate cells, isoflavones mostly stimulate ERβ and therefore suppress cell proliferation and promote differentiation [98].

##### Androgenic and Antiandrogenic Effects of Isoflavones

Isoflavones act on the enzymes 5-alpha reductase (5αR) and aromatase. Genistein inhibits 5αR activity in prostate fibroblasts, at a high dose, with an IC (50) of 710 μM [99].

At high doses genistein also inhibits some of the enzymes involved in T synthesis, so it can act as an antiandrogen. In the fetal murine testis, at a dose of 10 nM (corresponding to a dose of 100 mg/kg in humans), genistein reduces the early synthesis of T, via an ERα-dependent mechanism [100]. In rats in the pubertal stage, administration of genistein at a dose of 40 mg/kg was followed by a slight decrease in serum T, while a dose of 10 mg/kg had no effect on serum T. Interestingly, in castrated rats, genistein exhibits pronounced androgenic activity in the prostate gland [101,102].

**Figure 7:**
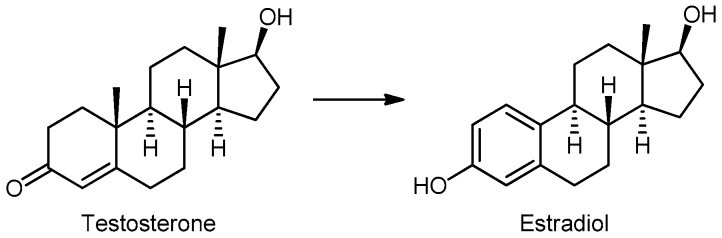
Aromatase converts testosterone to estradiol.

Isoflavones also act on the enzyme aromatase, or estrogen synthetase, which converts T to estradiol (E2) and androsterone to estrone (E1) (Figure 7). Genistein increases aromatase activity in ovarian, breast, and estrogen-dependent breast cancer cells, and may nullify the effects of anti-aromatase drugs in these cells, which should be taken into consideration in patients taking tamoxifen [103,104].

Genistein and D vitamin synergistically inhibit prostate epithelial cell growth. The flow cytometry showed that genistein induces arrest in the G (2) M phase, and vitamin D induces arrest in the G (1/0) phase of the cell cycle [105].

##### The Effects of Isoflavones on Prostate Cancer

Genistein has been previously known to block cell cycle progression in the G1 phase and inhibit PSA expression in PCa cell lines. At a dose of 1.25–10 μg / mL, genistein reduced the growth of BPH and PCa in histoculture. Physiological concentrations of genistein can reduce the expression of AR, mRNA, and protein levels in androgen-sensitive LNCaP cells [106,107].

A large number of in vitro studies have proven that genistein inhibits the growth and metastatic activity of hormone-dependent and hormone-independent PCa, with an IC50 of 5–40 nM (2–10 mg/mL). At doses ≤20 mM, genistein inhibits growth and induces apoptosis in LNCaP, DU-145, and PC3 lines. At 50 mM/L, genistein completely inhibits the expression of the androgen-responsive *PART-1* gene in LNCaP cells [108,109,110].

Chiyomaru found that genistein inhibits cell growth on PC3 and DU145 by down-regulating the oncogenic HOTAIR (human gene located on chromosome 12) molecule and altering the expression of several micro-RNAs [111].

More recent studies show that phytoestrogens do not act on AR but reactivate ERβ, which is otherwise “dormant” during carcinogenesis; so, reduced expression of ERβ leads to the same effect as androgen stimulation [112]. ERβ reactivation is accompanied by reduced expression of androgen markers, such as PSA and PCA3, the androgen coactivator PDEF and the IGF-1 receptor. Thus, activation of ERβ leads to an anti-androgenic effect on PCa [113,114]. In fact, genistein and ERβ work together to prevent PCa cell proliferation; genistein increases ER-β levels via reducing its promoter methylation, while ERβ mediates the preventive action of genistein [115].

Spagnuolo summarized the molecular mechanisms of genistein action: action on caspases, Bax (B cell lymphoma 2 (Bcl-2) -associated X protein), KIF20A (Bcl-2, kinesin-like protein 20A), ERK1/2 (extracellular signal-regulated kinase 1/2), NF-κB (nuclear transcription factor κB), MAPK (mitogen-activated protein kinase), IκB (inhibitor of NF-κB), Wnt/β-catenin (Wingless and integration 1 β-catenin), and PI3K/Akt (phosphoinositide 3 kinase/Akt) signaling pathways. Genistein works synergistically with adriamycin, docetaxel, and tamoxifen [92].

##### Animal Experiments

At a dose of 1 mg/g, genistein reduces prostate weight in rats and inhibits the expression of key cellular pathways. Kim believes that genistein at higher doses acts more as a tyrosine kinase receptor inhibitor than via the ERβ receptor [116]. Yang has demonstrated that soya isoflavones inhibit prostate hyperplasia and increase NO and NO synthase expression in rats [117]. Isoflavone-rich diets lead to a significant decrease in PCa growth and a decrease in the incidence of spontaneous PCa formation in mice and rats. When PCa-implanted rats are given subcutaneous injections of genistein (50 mg/kg), tumor growth is inhibited, which is not seen at doses appropriate to those in the human diet [118,119]. At massive doses of 250–1000 mg/kg, genistein decreases the expression of both AR and ER in the rat prostate [120].

##### Human Studies in Healthy Persons and BPH Patients

Healthy men consumed portions of 400 mL of soy milk, with 54 mg of genistein and 34 mg of daidzein. After eight weeks, no differences in E2, T, free T and SHBG (sex hormone-binding globulin) concentrations were observed between the persons and the control group. The only difference was the decrease in estrone concentration, in the milk-drinking group [121]. Busby showed no evidence of genistein toxicity or changes in blood biochemical analyses in healthy men taking doses of genistein up to 16 mg/kg body weight (over 1000 mg daily). The plasma half-life of genistein was 3.2 h and that of daidzein was 4.2 h [122]. Hong measured isoflavone levels in normal human prostate and BPH tissue after surgery. He found that the concentration of genistein in BPH was significantly lower than in healthy prostate (65 ng/mL vs 87 ng/mL) [123]. Wong administered 40 mg of isoflavone daily, or placebo to patients with BPH. The patients had excellent tolerance of isoflavones for 12 months, but they experienced very mild clinical improvement. Serum T level did not change after 12 months of isoflavone intake [124].

##### Human Studies in Prostate Cancer Patients

Numerous epidemiological studies have shown a lower incidence of PCa in Asia, where soya-based foods make up a large proportion of the diet [125,126]. Based on these studies, it was hypothesized that soya isoflavones may have a protective effect on PCa. This hypothesis is supported by the fact that in the USA, the incidence of latent PCa is twice as high as in Japan, but, the incidence of clinically manifest PCa is as much as 15 times higher, suggesting that there are some factors in Japanese men slowing or delaying the development of clinically significant PCa [127]. A soya-based diet could be one of these factors. Specifically, when migrating to the USA and changing their diet, Asians have the same rate of PCa as other Americans, with the rate of illness correlating positively with immigration time. Hawaii residents who are Japanese, consume soya products and have a lower risk of PCa [39,128].

In 1998, the results of a multinational study from 42 countries showed that the use of soya products significantly reduced the mortality of PCa [129]. However, it is difficult to prove the therapeutic effect of genistein on humans, due to the different study design, the small number of patients, the short treatment period, and the lack of standardized active ingredient formulas [130]. In a 2014 meta-analysis, two studies confirmed that there was a potential role of soy isoflavones on PCa reduction, but data from other studies were inconsistent [131].

A meta-analysis of 11 different studies (2015) showed that high levels of genistein and daidzein and elevated serum enterolactone concentrations were associated with a significantly reduced risk of developing PCa [132]. Russo (2017) found that genistein use was associated with a decreased risk of PCa. An incidental finding was that the use of lignans was associated with an increased risk of PCa disease [133].

Jarred found significantly higher tumor cell apoptosis in patients taking 160 mg of isoflavone extract before surgery, i.e., radical prostatectomy (RP) [134]. Fischer administered soy preparations to patients with PCa with 300 or 600 mg of genistein and 150 or 300 mg of daidzein for 12 weeks. He found a 32% decrease in serum DHT levels and mild estrogenic effects, such as changes in the chest, or hot flushes [135]. DeVere White gave patients with PCa who refused active treatment, or relapsed, an extract of 450 mg genistein +450 mg other aglycone isoflavones daily, for six months. A significant decrease in PSA was observed in all patients [136].

Van Veldhuizen administered isoflavones, at a dose of 112 to 224 mg daily, to patients prior to RP. After surgery, the values of serum T, E2, and expression of ERα receptors were compared in preoperative biopsied tissue and definitive histopathological findings. A mild decrease in serum T level was observed in most patients. There was also a decrease in serum E2 as well as decreased expression of ERα in tissue [137].

Jarrard gave patients before RP genistein, 600 mg daily. The patients also received a single dose of 200,000 IU of vitamin D3, at the beginning of the research. The increased AR expression was demonstrated relative to the placebo group. It was shown that vitamin D inhibited the proliferation of the prostate epithelium, and genistein enhanced this effect by inhibiting the CYP24 enzyme, which is responsible for the intracellular metabolism of vitamin D. Both genistein and vitamin D inhibited the synthesis of important mediator of the inflammation in prostate, prostaglandin E2 [138].

#### 3.5.5. Flavanols (Flavan-3-ols) or Catechins

**Figure 8:**
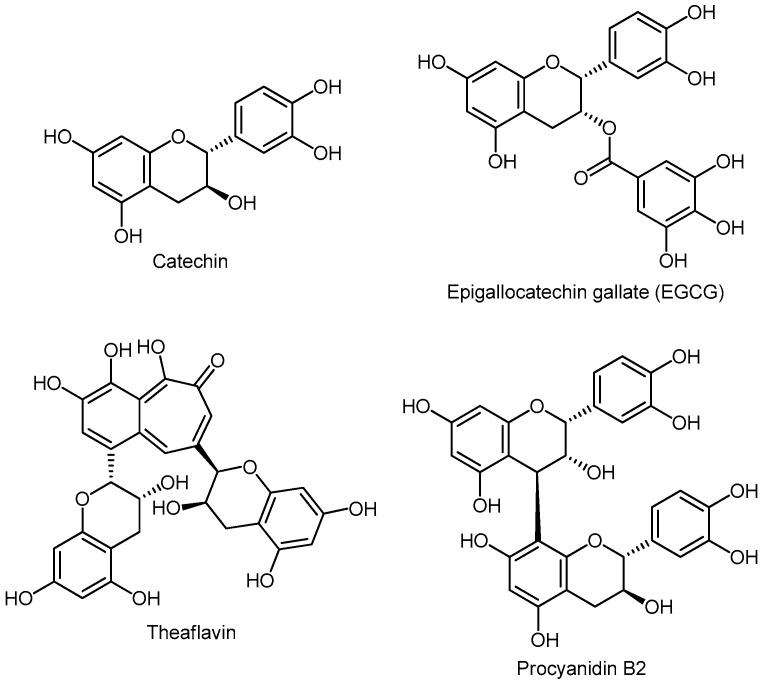
Chemical structures of some flavan-3-ol derivatives.

Flavan-3-ols are found in large quantities in tea, wine, bananas, apples, blueberries, peaches, and pears. These compounds include catechin, epicatechin, epicatechin-3-gallate, epigallocatechin and epigallocatechin-3-gallate, proanthocyanidins, theaflavins, and thearubigins [139]. Chemical structures of some flavan-3-ol derivatives are presented in Figure 8. Flavanols are divided into two subclasses: a) monomers (like catechin) and b) oligomers (proanthocyanidins). The catechin (monomer) molecule is a building block of polymers (proanthocyanidins) and higher-order polymers (anthocyanidins). Catechin monomers, dimers, and trimers are colorless. Higher-order polymers, anthocyanidins, have a red color and turn into tannins. Catechins are found in abundance in tea plants, cocoa plants, chocolates, fruits, vegetables, and wine [140,141].

##### Monomers

The flavan-3-ol monomers are catechin, epicatechin (EC), epicatechin-3-gallate (ECG), epigallocatechin (EGC), and epigallocatechin-3-gallate (EGCG). All listed catechins are present in a high concentration in green tea (30–42%) and less, in black tea (3–10%) [142]. The positive effects of catechins on human health are likely due to the chelation of metal ions such as iron (Fe) and copper (Cu). The iron and phenolic compounds in the gut lumen during digestion, as well as Fe-chelating complexes between Fe and polyphenols, have been demonstrated [143,144]. Some authors believe that the intake of six cups of green tea a day inhibits the carcinogenesis of PCa.

After digestion in the human digestive tract, the fate of the complex of low-molecular-weight phenolic compounds and proteins is not entirely clear. In an in vitro digestion model, Laurent found that about 44% of catechins and 85% of epicatechins disappear after two hours of intestinal incubation, probably due to their interaction with pancreatic enzymes [145].

In Vitro Studies

Catechins from green tea cause cell cycle arrest and apoptosis in malignant cells; EGCG is the most potent catechin that inhibits cell growth and causes apoptosis in human PCa lines. In addition, EGCG was proven to inhibit the activity of the enzyme NO synthase [146,147,148].

Agarwal found that in DU145 cells genistein and EGCG caused inhibition of erbB1 membrane receptor activation caused by TGFα. Silymarin, genistein, and EGCG caused inhibition of cell growth at lower doses and complete growth inhibition at high doses [51]. Gupta has demonstrated that EGCG-induced apoptosis in LNCaP cells is performed by two pathways: the first is the stabilization of tumor suppressor gene *p53* and the reduction of MDM2 protein expression; the second pathway is the negative regulation of NF-jB activity, which leads to a decrease in the expression of the anti-apoptotic protein Bcl-2. The *p53* gene is the most commonly mutated gene in PCa and other human cancers. NF-jB is a nuclear transcription factor that controls various immune responses, such as inflammation and apoptosis [149,150].

In PCa cells, EGCG increases the expression of CDK inhibitors, such as *p16*, *p18*, *p21*, and the expression of *p53* gene. In addition, EGCG has been shown to stabilize the *p53* gene and thus induce apoptosis in LNCaP cells [150,151]. Moreover, it was demonstrated that EGCG induces gene expression for 16 kinases and phosphatases in prostate cells, including the tumor suppressor gene (*SHP-1*) and the prostatic acid phosphatase-producing genes [152].

On DU145 and LNCaP cell lines, Siddiqui has demonstrated that EGCG causes induction of apoptosis as well as cell cycle arrest in the G0/G1 phase [153]. On LNCaP cells, EGCG has been shown to reduce PSA production; a decrease in PSA production was associated with the length of EGCG administration [154].

Animal Studies

Liao inoculated cells from PC-3 and LNCaP 104-R (androgen-repressed) lines into nude mice. Intraperitoneal injection of EGCG inhibited growth and rapidly reduced the size of human PCa in nude mice [155]. Kao reported that plasma T concentrations decreased by 70% in EGCG treated mice [156]. Hipaka showed in 2002 that EGCG and ECG inhibit 5αR activity in rats [157]. In guinea pigs receiving catechin, quercetin, and resveratrol, aortic fat reduction of 84%, 80%, and 76%, respectively, was found compared with the control group [158].

Human Studies

Tea polyphenols are partially resorbed from the intestinal tract so that they can exert effects not only in the gut but throughout the body [159]. Numerous epidemiological studies have shown that Japanese and Chinese inhabitants who regularly drink tea have a lower incidence of PCa, and that the risk of PCa decreases with increasing frequency, duration, and amount of green tea drinking [160]. Jain has shown that people in Canada who drink over two cups of tea a day have a lower incidence of PCa [161].Yang found that after taking 1.5 g of green tea dissolved in 500 mL of water, the maximum plasma concentration (Cmax) of EGCG was 326 ng/mL, EGC, 550 ng/mL, and EC, 190 ng/mL. When the dose was increased to 3.0 g, Cmax increased three times. The half-life of EGCG (5.0–5.5 h) was higher than the half-life of EGCG or EC (2.5–3.4 h) [162]. Catechin levels in saliva after drinking a green tea were significantly higher than in plasma, but with a shorter elimination half-life than in plasma, of 10–20 min. The catechin concentrations in saliva were: EGC; 11.7–43.9 µg/mL, EGCG; 4.8–22 µg/mL, and EC; 1.8–7.5 µg/mL. However, keeping the tea in the mouth for several minutes without ingestion caused higher concentrations of catechins in saliva and EGC in urine. These results suggest that EGCG is converted to EGC in the oral cavity and that both catechins are absorbed through the oral mucosa. Slow drinking of tea is important for catechin intake [163]. Pisters found that a dose of 1.0 g/m2 green tea extract, three times a day, was well tolerated and did not cause side effects. This dose was equivalent to 7–8 Japanese cups of 120 mL green tea [164].

However, in a major European study (2018), involving 140000 subjects over a 14 year period, no relationship was found between coffee and/or tea consumption and the risk of PCa [165].

##### Oligomers

Proanthocyanidins are blue and purple pigments found in numerous plants. A large number of these compounds are dimers of epicatechin (procyanidin A1, procyanidin A2) and oligomers of catechins and epicatechins and their esters with gallic acid. Proanthocyanidins are found in a large number of plants, such as apple, cinnamon, aronia, coconut, grapes and grape products, blueberries, cranberries, black currants, and green and black tea. The highest amount of proanthocyanidins is found in chocolate and apple (165 mg and 147 mg, per serving), significantly higher than in red wine and cranberry juice (22 mg and 32 mg per serving) [141].

Since the discovery of “French Paradox”, polyphenols from grapes and wine have attracted the attention of scientists. The average concentration of polyphenols in certain parts of the grapes, expressed as gallic acid equivalent (GAE) is: in seeds 2180 mg/g, in skin 375 mg/g, in leaf 350 mg/g and in “meat”, i.e., the juicy part, only 24 mg/g [166].

Some phenolic compounds in grapes are bioavailable, while compounds composed of large molecules are not, because they cannot be absorbed [167]. Of the phenolic compounds, the grapes contain mainly flavan-3-ols, flavonols, anthocyanins, stilbenes, and phenolic acids. About 60–70% of polyphenols in grapes are present in grape seeds, as procyanidins, that is, as dimers, trimers and other oligomers of flavan-3-ols [168]. Flavan-3-ols are present in large quantities in white grapes, while pigments, and anthocyanins, are mainly found in the skin of red grapes. Both catechin monomers and dimers have a high antioxidant capacity [169,170].

In Vivo Studies

Kaur found that grape seed extract (GSE) had a higher antioxidant capacity than vitamins C and E. At a concentration of 100 mg/L, GSE inhibited superoxide anion and hydroxyl radical by about 80%, which was significantly higher than with vitamin C (~15%) and vitamin E (~40%) [171]. In DU145 cells, commercial GSE extract with 95% procyanidins, induced apoptosis and inhibited the EGFR-Shc-ERK1/2-Elk1-AP1 signaling pathway [172,173]. Singh demonstrated that GSE had anticancer activity against hormone-refractory PCa (HRCaP) [174]. Schmidt found that blueberry proanthocyanidins inhibited the growth of LNCaP cells [175]. Baud demonstrated that GSE inhibits the NF-kB pathway and thus induces apoptosis in DU145 cells [176].

After two hours of incubation in vitro, the monomers and dimers were stable in the intestinal medium at pH 7; dimer degradation began at pH 7.4 and all dimers disappeared at pH 8.5. At pH 7.5, after two hours of incubation, 15–34% of epicatechins underwent degradation while catechin was stable [177]. In an in vitro digestion experiment, Argyri showed that red wine reduced the concentration of digested phenolic compounds, due to the formation of Fe-polyphenolic chelates. Also, consuming red wine reduces the oxidative stress induced by Cu-oxidized LDL and increases the concentration of HDL cholesterol; similar to wine, grape juice also inhibits Cu-induced oxidation of human LDL [178,179].

Animal Studies

Chokkalingam has demonstrated that GSE increases the level of insulin-like growth factor binding protein-3 (IGFBP-3), which can influence the suppression of PCa growth. DU145 cells were implanted to nude mice; after seven weeks of feeding with GSE, there was a decrease in the proliferation index and an increase in the apoptosis index, as well as a decrease in tumor size. GSE strongly inhibited the secretion of vascular endothelial growth factor (VEGF) and strongly increased IGFBP-3 production in the tumor [180].

Human Studies

Grape polyphenols are rapidly absorbed into the plasma, reaching a maximum concentration 2–3 h after ingestion [181]. After two weeks of daily consumption of red wine (375 mL), an increased concentration of phenolic compounds in the plasma was recorded [182].

Polyphenols from red wine and grape juice inhibit LDL oxidation and increase HDL concentration, which reduces the risk of atherosclerosis and coronary heart disease. Grape polyphenols also have a beneficial effect on diabetes by improving insulin resistance. Moderate and regular consumption of red wine reduces the risk of type 2 diabetes by 30% [183,184,185]. In a 2013 review, Vance stated that coffee and tea provide protection against advanced PCa. Lycopene seems to reduce a disease risk of PCa, while selenium, vitamin C, and beta-carotene do not have such evidence. Regarding vitamin E, alpha-tocopherol potentially increases the disease risk from PCa, and gamma-tocopherol reduces it [186].

#### 3.5.6. Anthocyanins

Anthocyanins (anthos = flower, kianeos = dark blue) are water soluble pigments that, can be red, blue, or purple, depending on pH. About 2% of all hydrocarbons generated during photosynthesis are converted to flavonoids and their derivatives, such as anthocyanins, which have antioxidant properties and provide protection against ultraviolet radiation and cold [187].

Anthocyanin analogs without sugar are called anthocyanidins. Anthocyanins are found in all tissues of higher plants, especially in fruits, flowers, and leaves. They are mainly found in fruits such as cranberries, black currants, red and merlot grapes, strawberries, blackberries, blueberries, raspberries, cherries, red apples, red peaches and vegetables, like eggplant, black rice, and red cabbage [188].

**Figure 9:**
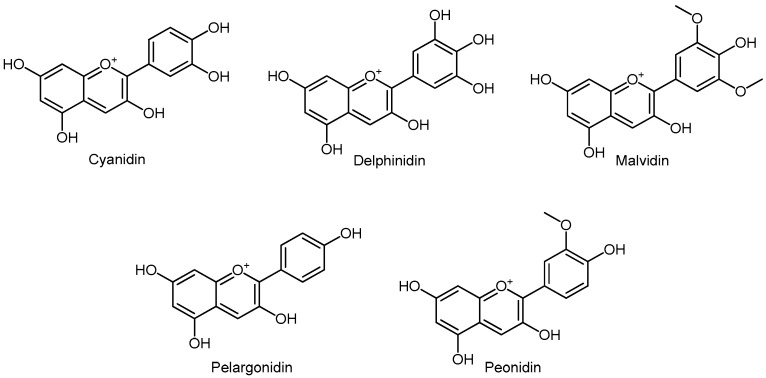
Chemical structures of most common anthocyanins found in nature.

The highest concentrations of anthocyanins per 100 g of fruits are found in the shell of black soybean (2000 mg), aronia (1480 mg), red grapes (888 mg), and blue eggplant (750 mg) [189,190]. Today, 200 different anthocyanins are known; only in red grapes, there are over 15 anthocyanins. The most common anthocyanins in nature are cyanidin, delphinidin, malvidin, pelargonidin, and peonidin (Figure 9), in the form of glycosides.

In the wine, malvidin-3-glucoside has the highest antioxidant capacity [191]. The most abundant anthocyanin in the diet is cyanidin-3-*O*-β-glucopyranoside (C3G), especially abundant in red orange juice. The anti-proliferative effect of C3G via activation of caspase-3 and induction of p21 protein expression in two different lines, LNCaP and DU145, has been demonstrated [192]. In another study, the anthocyanin treatment of DU-145 cells resulted in a significant increase of apoptosis and a significant decrease in p53 and Bcl-2 expressions and also a significant decrease in PSA and AR expressions. In addition, the anthocyanin treatment significantly inhibited tumor growth in the xenograft model [193].

Anthocyanins are poorly absorbed in the human gastrointestinal tract, and massively metabolized by the intestinal microflora into several metabolites, such as phenolic acids 3-*O*-methyl-gallic (Megal), gallic (Gal), syringic, vanillic, and protocatechuic acid. The digestion of anthocyanins into the colon is completed six hours after their administration. Megal and Gal are effective in reducing the vitality of colon cancer cells. Anthocyanins from wine and grapes inhibit phosphodiesterase-5 activity, lead to vasorelaxation and thus reduce the risk of cardiovascular diseases [194,195,196].

Although the effects of anthocyanins have been demonstrated in vitro, there is no reliable evidence that anthocyanins act as antioxidants after consuming these plants. The fate of anthocyanins in vivo shows that they are poorly conserved and that most of the chemically modified metabolites are rapidly excreted. However, a 2019 study proved that the administration of anthocyanins caused an increase in organ antioxidant capacity in animal models [197].

The results of a 20 year study (2012) showed that high anthocyanin intake reduced the risk of T2D [198,199].

### 3.6. Phenolic Acids

#### 3.6.1. Hydroxybenzoic Acids

**Figure 10:**
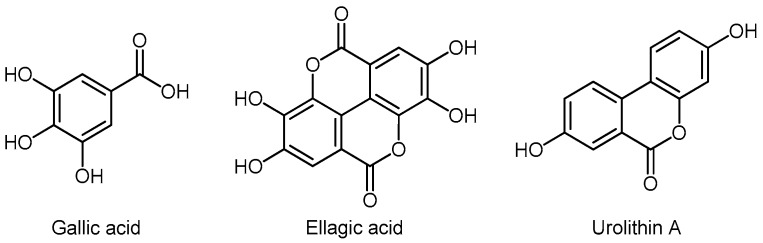
Chemical structures of gallic acid, ellagic acid, and urolithin A.

Gallic acid (GA) is naturally found in grapes, tea, hops, and oak bark. In nature, GA is free or as a part of soluble tannins. Gallic acid produces dimers, such as ellagic acid (EA) (Figure 10). During hydrolysis, the soluble tannins break down into GA and glucose (gallotannins), or to EA and glucose (ellagitannins). Plants produce EA by the hydrolysis of tannins, such as ellagitannin. In humans, EA derivatives are processed in the intestine by the action of the intestinal microflora to form urolithins (Figure 9). The largest amount of EA is found in nuts, berries, grapes, peaches, and pomegranates, while ellagitannins are most common in fruits and leaves of raspberries, blackberries, blueberries, nuts, and red grape [200,201].

Kaur demonstrated that GA leads to apoptosis in DU145 and 22Rv1 cell lines. In nude mice fed with GA, tumor growth inhibition occurs [202]. It has also been shown that GA reduces survival, proliferation, and invasion in PC3 cells [203]. In a 2015 study, Park demonstrated the apoptotic effect of gallotannin in DU145 and PC-3 cell lines. In addition, gallotannin decreased the expression of some survival genes, such as *Mcl-1*.The inhibition of *Mcl-1* and caspase activation are the key moments in PCa cell apoptosis, induced by gallotannin [204].

Dimeric ellagitannin, Enotein B, causes a decrease in LNCaP cell proliferation and reduction of BPH in rats. Enotein B is found in the willowherb (*Epilobium angustifolium*), which is used in traditional medicine to treat BPH and prostate inflammation [205]. In human volunteers who drank willowherb tea, only urolithin conjugates were found in urine. In a 2017 paper, Roberts examined the metabolism of ellagitannins (ET) in humans. In the usual diet, the average daily intake of polyphenols was about 1250 mg, and coffee and tea were the main sources of polyphenols (1100 mg daily), and provided the intake of about 12 mg ET daily [206].

A significant amount of ET is found in pomegranate (*Punica granatum*). There is evidence that pomegranate has a positive effect on the treatment and prevention of diabetes, atherosclerosis, PCa, breast, colon, lung, and skin cancers [207,208].

Ellagitannins from pomegranate called punicalagin (PN) caused induction of apoptosis in PC-3 and LNCaP cells [209]. In addition to ellagitannins, large amounts of anthocyanins are also found in the pomegranate. Pomegranate fruit, as well as pomegranate juice, extract, and oil, have potent antioxidant activity and antitumor properties in various tumors, including PCa [207].

##### Urolithins

Urolithins A and B are metabolites of the microflora of the colon produced by the intake of EC derivatives. Sánchez-González (2014) has shown that foods rich in ET, such as nuts, can contribute to the prevention of PCa. In the study, urolithins induced apoptosis in LNCaP cells and this effect was correlated with a decrease in Bcl-2 protein levels. Urolithins most likely block the expression of the androgen receptor (AR), and thus, leads to the induction of apoptosis and decreased PSA synthesis [210].

#### 3.6.2. Hydroxycinnamic Acids

**Figure 11:**
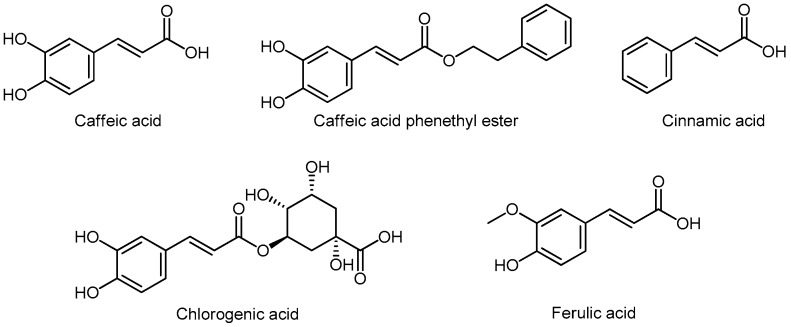
Chemical structures of some hydroxycinnamic acids.

Chemical structures of the most important hydroxycinnamic acids are presented in Figure 11. Caffeic acid is found in burdock, hawthorn, artichoke, pear, apple, oregano, basil, and thyme. Caffeic acid and its natural ester-caffeic acid phenethyl ester (CAPE) are potent inhibitors of the androgen-dependent PCa lines [211]. Liu demonstrated that caffeic acid and CAPE from bee propolis [212] suppressed tumor growth and Akt signals in human PCa cells and showed a synergistic effect with chemotherapeutics and external radiation [213]. Cinnamic acid is found in the aloe plant. Esters of cinnamic acid strongly inhibit the growth of prostate and breast cancer by inducing apoptosis [214]. Chlorogenic acid is found in *Echinacea*, strawberries, pineapple, coffee, sunflower, and blueberries. In the animal model, chlorogenic acid inhibits BPH growth, probably via inhibition of 5αR [215]. Ferulic acid is found in oats, rice, artichokes, oranges, pineapples, apples, and peanuts. In a 2015 paper, Eroglu demonstrated that ferulic acid caused cell cycle arrest in PC3 cells and apoptosis in LNCaP cells [216].

### 3.7. Stilbenes

**Figure 12:**
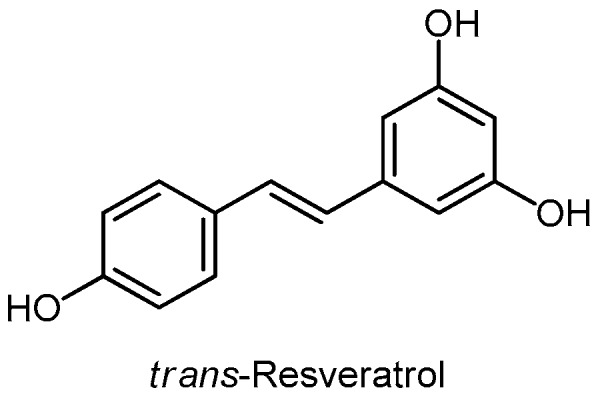
Chemical structure of *trans*-resveratrol.

Resveratrol (Figure 12) is a powerful antioxidant found mainly in grapes and red wine, and in berries and onions. The poor hydro-solubility and bioavailability of resveratrol are limitations of its clinical use. Cheng (2017) found that resveratrol can induce nuclear accumulation of COX-2 (Cyclooxygenase-2) and thus enhance p53-dependent inhibition of antiproliferation in cancer. The enzyme COX-2 is involved in the development of cancer. Treatment of LNCaP cells with resveratrol led to the phosphorylation and the nuclear translocation of ERK1/2 (mitogen-activated protein kinase) and the accumulation of nuclear COX-2 and subsequently to the complex formation with pERK1/2 and p53 [217].

Saralkar (2017) believes that resveratrol will be able to be used in nanoparticles in the future. In fact, hydrophobic medicines can be “trapped” in nanoparticles, such as Ca-alginate. Nanoparticles filled with medicines have had a cytotoxic effect on the cells of DU-145. It is believed that the nanoparticles with Ca-alginate could probably be given intravenously [218].

### 3.8. Lignans

**Figure 13:**
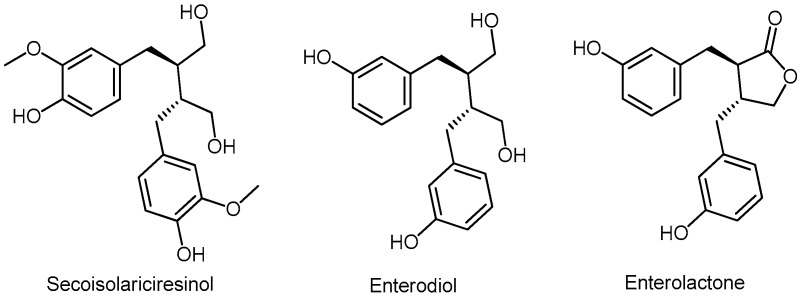
Chemical structure of the most important plant lignans.

The highest concentration of lignans is found in oilseed rape (20 µg/100 g) [219]. The most important plant lignan, secoisolariciresinol diglucoside (SDG), is an antioxidant phytoestrogen found in flax, sunflower, sesame and pumpkin seeds. In the human gastrointestinal tract, SDG is further metabolized to secoisolariciresinol, enterodiol, and enterolactone (ENL) (Figure 13) [220].

The flax extract has been shown to prevent the induced BPH in rats [221]. Ren (2016) showed that enterolactone binds to G protein-coupled estrogen receptor 1 (GPER) and thus reduces the prostate growth in rats. When estrogen binds to GPER, rapid, non-genomic signaling occurs in the cell [222]. De Amorim Ribeiro (2017) gave flax-based food to rats with induced BPH, for 20 weeks. The flax was found to have a protective effect on prostate epithelium in previously induced BPH [223].

The evidence from preclinical and animal studies has demonstrated the anticancer action of flax lignans, especially the action of enterolactone against PCa. However, after the ingestion of flax through food, rapid metabolism of lignans occurs, so that only glucuronic acid conjugates, which have modest effects in vivo [224], considering the systemic circulation.

A recent Swedish study of 1010 cases during a mean follow-up of 14.6 years found that plasma ENL concentration was positively associated with consumption of high-fiber bread, fruit, tea, and coffee, while it was negatively associated with smoking and obesity. In subgroup of men with abdominal obesity, the association between high ENL levels and lower odds of high-risk PCA was proved [225].

### 3.9. Curcumin

**Figure 14:**
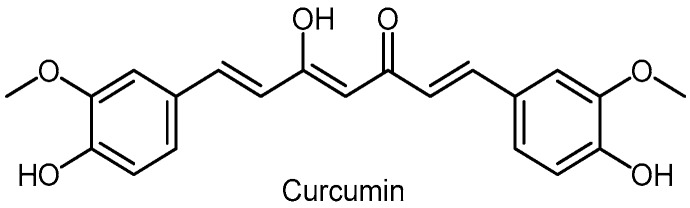
Chemical structure of curcumin.

Curcumin (diferuloylmethane) is a consistent part of turmeric, a spice to give it the yellow powder color of curry (Figure 14).

PCa resistance to a therapy is associated with the abnormally increased expression of Bcl-2, and androgens receptor (AR), epidermal growth factor receptor (EGF), HER2 (human epidermal growth factor receptor 2), cyclic D1, and cyclone-oxygenize (COX-2), and all of them are connected to the activation of the transcription factor NF-kB. Nuclear factor-kB (NF-κB) is a protein complex and it controls DNA transcription, cytokine production, and cell survival. NF-κB is placed in cells of almost all animals and it is included into the cell answer to stress, cytokines, free radicals, heavy metals, ultraviolet sunrays, oxidized LDL, and also to bacterial and virus agents [52].

The agent that can slow down these mechanisms would be good both for the prevention and the treatment of PCa. Curcumin can modulate several of these ways. It has been shown that curcumin to sublimates the proliferation of androgen-dependent lines, such as LNCaP, but also of androgen-independent lines, such as DU145. Besides this, it has been shown for curcumin to increase the sensitivity of cell cultures of PCa onto gamma-radiation. Curcumin reduces the *trans*-activation and the expression of AR and it acts as its antagonist. Thus, curcumin reduces the expression of receptors of EGF and induces the degradation of HER2. Curcumin reduces angiogenesis in vivo and the expression of VEGF. The pathway of t PI-3K/AKT plays a critical role in the cell activation of CaP. The phosphatase regulating this pathway, PTEN, is mutated with PCa and it leads to AKT activation studies on the ability of curcumin to inhibit AKT activation AKT [226].

Mohebbati et al. have come to the facts by researching the data banks for curcumin as an inhibitor of the tumor necrosis factor production (TNF-α) and prostaglandin E2 (PGE2), but it increases the caspase activity (3, 8 i 9) in HL-60 prostate carcinoma. Besides this, we have found for curcumin to sublimate vascular and endothelial growth factor (VEGF) in the line of carcinoma ovarium [227].

In a study in 2018, Chen examined the anti-carcinoma potential of curcumin analogs of the second generation. The cells of PC3 and DU145 were treated in series of curcumin analogs, in concentrations of 0–10 μM. It was found for curcumin to inhibit the expression of the nuclear factor (NF)-kB, mTOR (mammalian target of rapamycin), AKT and p-AKT. For curcumin, it was found to have anti-carcinoma effect against CRPC cells [228].

The aim of the investigation of Morabito and co-workers was to verify whether the different concentrations of curcumin, conducted on human erythrocytes, may counteract Band 3-mediated anion transport alterations due to oxidative stress. This study demonstrated that curcumin at a concentration of 10 uM was very effective in protecting red cells from oxidative stress. [229].

In the paper in 2018, Ide et al. examined the effect of curcumin on the intracranial synthesis of androgen in the cells of CaP, in the lines of LNCaP and 22Rv1. The tissue from the trans-gene adenocarcinoma of the mouse prostate (TRAMP) was taken after one month of oral intake of 200 mg/kg/curcumin per day. Curcumin reduced the expression of steroid acute regulatory proteins CYP11A1 and HSD3B2, and it confirmed a reduction of testosterone production. At the same time, it confirmed the testosterone reduction in the prostate tissue of TRAMP mice. Simultaneously, curcumin brought to the increased level of Aldo–Keto reduction 1C2 (AKR1C2), and it confirms reduced level of dihydrotestosterone [230].

## 4. Conclusions

It is fascinating how much biochemical processes in plants are incorporated into the metabolism of animal cells. However, this fact is logical, when we know how many billion years a common microcosmos has existed in plant and animal cells. Herbivores have used plant nutrients in their diets for eons. However, in addition to sugars, proteins, and fats, animals also ingest thousands of secondary plant metabolites, such as polyphenols. Polyphenols in plants play important roles such as protection against herbivores, oxidative stress, UV radiation, etc. However, their roles in animal and human organisms are much too complicated and plentiful to be completely understood.

There are a very large number of investigations on human cell cultures and animal models to confirm the antioxidant and antitumor effects of polyphenols. In addition, extensive epidemiological studies and centuries-old traditional folk experiences confirm the positive effects of polyphenols on human health. However, the pathways of polyphenols in the human body are difficult to follow, due to their relatively low resorption, the production of a large number of metabolites and relatively rapid excretion from the body.

The future researches on the vast range of polyphenolic compounds in nature and their effects on the human body will probably evolve towards increasing resorption and bioavailability, through nanotechnology or other technologies. In the future, major randomized epidemiological studies will provide more comprehensive answers to the questions of the role of polyphenols in human health.
